# Editorial: Sexual Dimorphism of the Immune Inflammatory Response in Infectious and Non-infectious Diseases

**DOI:** 10.3389/fimmu.2019.00107

**Published:** 2019-02-05

**Authors:** Mustapha Chamekh, Georges Casimir

**Affiliations:** ^1^Inflammation Unit, Laboratory of Pediatric Research, Faculty of Medicine, Free University of Brussels (ULB), Brussels, Belgium; ^2^Laboratory of Pediatric Research, Faculty of Medicine, University Children's Hospital HUDERF, Free University of Brussels (ULB), Brussels, Belgium

**Keywords:** gender dimorphism, inflammation, infectious diseases, chromosome X-linked genes, sexual hormones

With the emergence of the concept of personalized medicine, it is particularly interesting and important to uncover the mechanisms behind the differences observed in the physiopathology of many human diseases between males and female. Sex-driven differences in the type and magnitude of the immune inflammatory response during infection, trauma or vaccination are well recognized. Although recommendations to include gender as a biological variable in clinical and experimental animal studies are increasingly considered over the last years (1), still there is a dominance of male research subjects in the biomedical and experimental study design. The mean legitimate reason of these imbalances is to overcome the variability issues potentially attributed to the estrous cycle. Sexual hormones can indeed influence the outcomes. Yet, in many trauma and infectious situations, sexual dimorphism occurs in all age groups, including pre-pubertal infants, and therefore can not be fully explained by sexual hormones. There are several reports pointing the role of sex-specific genetic architecture with X-chromosome-linked genes playing a prominent role. Indeed, one of the X chromosomes is randomly inactivated in females early in embryogenesis to unsure the dose compensation with males. Consequently, females have mosaic cells expressing two X-linked gene alleles, giving them the great advantage to cope with genetic diseases associated with recessive mutations occurring on the X chromosome. Moreover, about 15% of X-linked genes escape inactivation and 10% have variable degree of inactivation, which may lead to the over-expression of some X-linked genes in females (2–4).

In the current Research Topic, review articles provide insights into the underlying mechanisms, discuss some of the encountered challenges and highlight gaps and perspectives. The article by Spolarics et al. is a very interesting overview on the role of chromosome X-linked genetic architecture in sex-related differences in the innate immune response. Of note is that many genes playing a key role in immune responses, such as IRAK-1, NOX2, and CD40 ligand, are located on the chromosome X. The authors elaborate the possibility that multiple chromosome X-linked genetic mechanisms including differences in chromosome X regulation and inheritance patterns between males and females and abundant polymorphism of X-linked immune genes may result in sex-related differential outcomes in infection and trauma.

Regarding pathogen-induced inflammation, it is widely established that the disease severity is sexually dimorphic with males generally more susceptible to acute infections than females. The later are, however, more prone to develop robust and persistent immune responses that may come at the cost of damaging chronic inflammation. For example, in HIV-1 infection model, the systemic immune activation being established as a strong predictor for disease progression to AIDS, is commonly higher in women than in men. Ziegler et al. summarize here recent findings on sex differences in HIV-1 infection with a focus on the role of chronic stimulation of type I interferons in increased levels of immune activation in women compared to men. The immune inflammatory response triggered upon microbial infection is host beneficial when kept under tight control, so the balance between protective vs. excessive and pathological inflammation is critical for the disease outcome. In another example of viral infection models, Coxsackievirus B3, known to cause more severe heart muscle inflammation in males than in females, Koenig et al. address the role played by steroid hormones during infection in mice. Notably, androgens promote pro-inflammatory immune responses and poor outcome in males, while estradiol promotes immune regulation that favors a protective response in females. Similarly, several clinical and experimental studies have showed that the severity of inflammatory symptoms in pneumonia vary between males and females according to the pathogen species. In this sense, the review by Chamekh et al. summarizes the current knowledge about sex-specific differences in infectious respiratory models and provides an up-date on selected immune genes like TLR and X-linked microRNAs that could play a key role in modulating differentially the immune inflammatory response in males and females.

Sexual dimorphism of the immune response has been reported in cancer as well. With the recent development of the immune checkpoint inhibitors that reverse the tumor-induced tolerance, there is a growing need to consider gender-based immunotherapy for optimizing cancer control. Capone et al. elaborate a perspective view on this aspect with a particular focus on the potential role of type I IFN-dependent signaling. Sex differences occur also in non-infectious chronic inflammatory disease such as asthma, cystic fibrosis or sickle anemia. The review by Laffont et al. covers the current understanding of the impact of sex hormones in allergic asthma which is mediated by exacerbated type 2 immune responses. A particular focus is made on the development and function of type 2 innate lymphoid cells that play a key role in the initiation of allergic responses through producing type 2 cytokines.

Inflammation can be considered as an active response aiming to avoid dramatic stress challenges to homeostasis. Interestingly, acidosis is observed in numerous acute inflammatory responses such as sepsis, therefore acid-base balance could be considered among the mechanisms that are crucial to cell homeostasis. Casimir et al. address in this Research Topic the impact of gender differences in acid-base balance on sex bias of inflammation and highlight the potent role that could be played by the endothelial cells in this process. On the other hand, growing evidences point toward the contribution of the gut microbiota in shaping the immune system and in health and disease in general. In the light of recent studies in human and mice showing that differences in gut microbiota between males and females exist, Elderman et al. present a review synthesis discussing the possibility that sex-dependent composition of microbiota could have a direct or an indirect influence on development of dimorphic immune response.

In conclusion, the reports presented in this Research Topic exemplify not only the prevalence of sex-dependent variations in infectious and non-infectious inflammatory diseases but also the complexity and multiplicity of the mechanisms at work, hence stressing the need for further studies to mainly uncover whether a uniform concept could be applied in each inflammatory setting.

## Author Contributions

All authors listed have made a substantial, direct and intellectual contribution to the work, and approved it for publication.

### Conflict of Interest Statement

The authors declare that the research was conducted in the absence of any commercial or financial relationships that could be construed as a potential conflict of interest.

## References

[1] Putting gender on the agenda Nature (2010) 465:665 10.1038/465665a 20535156

[2] Carrel L Willard HF. X-inactivation profile reveals extensive variability in X-linked gene expression in females. Nature (2005) 434:400–4. 10.1038/nature03479 15772666

[3] Tukiainen T Villani AC Yen A Rivas MA Marshall JL Satija R. Landscape of X chromosome inactivation across human tissues. Nature (2017) 550:244–8. 10.1038/nature24265 29022598 PMC5685192

[4] Libert C Dejager L Pinheiro I. The X chromosome in immune functions: when a chromosome makes the difference. Nat Rev Immunol. (2010) 10:594–604. 10.1038/nri2815 20651746

